# Identification of a 6-Cytokine Prognostic Signature in Patients with Primary Glioblastoma Harboring M2 Microglia/Macrophage Phenotype Relevance

**DOI:** 10.1371/journal.pone.0126022

**Published:** 2015-05-15

**Authors:** Jinquan Cai, Wei Zhang, Pei Yang, Yinyan Wang, Mingyang Li, Chuanbao Zhang, Zheng Wang, Huimin Hu, Yanwei Liu, Qingbin Li, Jinchong Wen, Bo Sun, Xiaofeng Wang, Tao Jiang, Chuanlu Jiang

**Affiliations:** 1 Department of Neurosurgery, The Second Affiliated Hospital of Harbin Medical University, Harbin, China; 2 Beijing Neurosurgical Institute, Capital Medical University, Beijing, China; 3 Beijing Institute for Brain Disorders Brain Tumor Center, Beijing, China; 4 Department of Neurosurgery, Beijing Tiantan Hospital, Capital Medical University, Beijing, China; 5 Chinese Glioma Cooperative Group (CGCG), Beijing, China; University of Michigan School of Medicine, UNITED STATES

## Abstract

**Background:**

Glioblastomas (GBM) are comprised of a heterogeneous population of tumor cells, immune cells, and extracellular matrix. Interactions among these different cell types and pro-/anti-inflammatory cytokines may promote tumor development and progression.

**Aims:**

The objective of this study was to develop a cytokine-related gene signature to improve outcome prediction for patients with primary GBM.

**Methods:**

Here, we used Cox regression and risk-score analysis to develop a cytokine-related gene signature in primary GBMs from the whole transcriptome sequencing profile of the Chinese Glioma Genome Atlas (CGGA) database (n=105). We also examined differences in immune cell phenotype and immune factor expression between the high-risk and low-risk groups.

**Results:**

Cytokine-related genes were ranked based on their ability to predict survival in the CGGA database. The six genes showing the strongest predictive value were CXCL10, IL17R, CCR2, IL17B, IL10RB, and CCL2. Patients with a high-risk score had poor overall survival and progression-free survival. Additionally, the high-risk group was characterized by increased mRNA expression of M2 microglia/macrophage markers and elevated levels of IL10 and TGFβ1.

**Conclusion:**

The six cytokine-related gene signature is sufficient to predict survival and to identify a subgroup of primary GBM exhibiting the M2 cell phenotype.

## Introduction

Glioblastoma (GBM) is a common and aggressive form of diffuse glioma, associated with short survival and uniformly fatal outcome, irrespective of treatment [1, 2]. Histologically, malignant gliomas are characterized by hypercellularity, nuclear pleomorphism, microvascular proliferation, and pseudopalisading necrosis [3]. Reactive gliosis, microglial activation, and disrupted vasculature are all common characteristics of gliomas [4]. Other disease hallmarks include breakdown of the blood-brain barrier (BBB) and increases in both hypoxia and necrosis [5]. Patients with primary GBM have a median progression-free survival of just over half a year and a median overall survival of 15–18 months; only a minority of patients shows median survival beyond 2 years [6]. Given these clinical challenges and the immune heterogeneity of glioblastoma, immunotherapy is an appealing treatment for these tumors [7–9].

The tumor microenvironment (TME) consists of tumor cells, immune cells, inflammatory cells, endothelial cells, and extracellular matrix [10]. Macrophages, specifically referred to as tumor-associated macrophages (TAMs), are the most common cell type among tumor-infiltrating immune cells [11]. TAMs from human neoplasms express arginase1, IL10, and transforming growth factor beta (TGFβ); these cytokines reduce the anti-tumor activity of T cells and natural killer cells and modulate tumor proliferation, infiltration, and angiogenesis [1]. In this study, we aimed to identify a cytokine-related signature based on mRNA expression profiling, which could divide glioblastoma patients into high-risk and low-risk subgroups with distinct clinical prognosis. Immunologic gene signatures, indicative of activated microglia, were enriched in the high-risk subgroup. Previous studies of TAM populations in glioma tissues have shown that activated microglia/macrophages (especially M2) express high levels of CD68, CD163, CD204, and CD206 [5, 12–14]. Based on these findings, we used these specific markers to identify the activation of macrophage phenotypes in tumor samples. Consistently, we also observed that myeloid-derived suppressor cell (MDSC) subset markers CD11b, CD14, CD15, and CD33 were also elevated in the high-risk subgroup. TGFβ and IL10 are produced by a wide variety of cells including M2 macrophages, type 2 CD4 T-helper cells, myeloid-derived suppressor cells (MDSC), a subset of CD8 T cells, mast cells, and CD4^+^CD25^+^Foxp3^+^Treg cells. Here, we find that both TGFβ and IL10 are implicated in malignancy of the high-risk subgroup tumors. These findings raise the possibility that treatment strategies targeting immunomodulatory cells infiltrating high-grade gliomas may be therapeutically useful.

## Materials and Methods

### Patients and Samples

Clinical information of 105 patients diagnosed with primary glioblastoma according to the 2007 World Health Organization (WHO) classification of tumors of the central nervous system [3] was obtained from the Chinese Glioma Genome Atlas (CGGA; http://www.cgga.org.cn) [15, 16]. Tumor tissue samples were obtained by surgical resection. All patients (age range: 18–81 years) provided written informed consent. The study was approved by the institutional review boards of Capital Medical University, the Second Affiliated Hospital of Harbin Medical University and Beijing Institute for Brain Disorders Brain Tumor Center, and written informed consent was obtained from all patients. Survival data were collected by clinics during patient visits and/or phone interviews. Patients who underwent biopsy alone were not followed up at our center and were therefore excluded from the survival analysis. The Cancer Genome Atlas (TCGA) database (n = 518) was used as the validation set (http://cancergenome.nih.gov) [17].

### Whole transcriptome sequencing

Total RNA was isolated using the RNeasy Mini Kit (Qiagen) according to the manufacturer’s instructions. A pestle and a QIAshredder (Qiagen) were used to disrupt and homogenize frozen tissue. RNA integrity was assessed using the 2100 Bioanalyzer (Agilent Technologies), and only high quality samples with an RNA Integrity Number (RIN) greater than or equal to 7.0 were used to construct the sequencing library. The subsequent steps included end repair, adapter ligation, size selection, and polymerase chain reaction enrichment. DNA fragment lengths were measured using a 2100 Bioanalyzer, with median insert sizes of 200 nucleotides. The libraries were sequenced on the Illumina HiSeq 2000 platform using the 101-bp paired-end sequencing strategy. Short sequence reads were aligned to the human reference genome (Hg 19 Refseq) using the Burrows-Wheeler Aligner (BWA, Version 0.6.2-r126) [18].

### Molecular analyses

#### Isocitrate dehydrogenase (IDH) mutations

Genomic DNA was extracted from frozen tissues with a QIAamp DNA Mini Kit (Qiagen) according to the manufacturer’s protocol. DNA concentration and quality were measured using a Nano-Drop ND-1000 spectrophotometer (NanoDrop Technologies, Houston, TX). Pyrosequencing of IDH1/2 mutations was supported by Gene-tech (Shanghai, China) and performed on a Pyro-Mark Q96 ID System (Qiagen, Valencia, Calif). The following primers were used for PCR amplification: IDH1 5’-GCTTGTGAGTGGATGGGTAAAAC-3’ and 5’-Biotin-TTGCCAACATGACTTACTTGATC-3’; and IDH2 5’-ATCCTGGGGGGGACTGTCTT-3’ and 5’-Biotin-CTCTCCACCCTGGCCTACCT-3’. The primer sequences used for pyrosequencing are 5’-TGGATGGGTAAAACCT-3’ for IDH1 and 5’-AGCCCATCACCATTG-3’ for IDH2 [19, 20].

#### O-6-methylguanine-DNA methyltransferase (MGMT) promoter methylation

MGMT promoter methylation status was assessed as previously reported [21]. Bisulfite modification of DNA was performed using the EpiTect Kit (Qiagen). Two primers were used to amplify the MGMT promoter region: 5’- GTTTYGGATATGTTGGGATA-3’ and reverse: 5’-biotin-ACCCAAACACTCACCAAATC-3’. The PCR analysis was performed in duplicate in 40μl reaction volume containing 0.5 μl of 10 μM each primer, 4μl 10× buffer, 3.2μl of 2.5 μM dNTPs, 2.5 U hotstart Taq (Takara, Madison, WI), and 2 μl of 10μM bisulfite-treated DNA. The PCR conditions were as follows: 95°C–3 min; 40 cycles of 95°C–15 s, 52°C–30 s, 72°C–30 s; 72°C–5 min (ABI PCR system 9700). DNA was purified from the total PCR product using QIAamp DNA Mini Kit (Qiagen) and then subjected to pyrosequencing (PyroMark Q96 ID System (Qiagen)) using the primer 5’-GGATATGTTGGGATAGT-3’ in accordance to the manufacturer’s instructions. The methylation values obtained were averaged across the seven CpG loci tested within the MGMT promoter. The GBM samples were considered MGMT promoter methylated with an average methylation of >10%.

### Statistical Analysis

RNA sequencing data was downloaded from the CGGA dataset. Gene expression was calculated using the RPKM method (reads per kilobase transcriptome per million reads) [22, 23]. The RPKM method eliminates the influence of varying gene lengths and sequencing discrepancies from the calculation of gene expression. Therefore, the calculated gene expression can be directly used to compare the differences in gene expression among samples [24].

To integrate cytokine-related gene sets, 821 cytokine-related genes were first extracted from canonical biological pathways in the Molecular Signatures Database v4.0 (MSigDB) (http://www.broad.mit.edu/gsea/msigdb/) [25, 26] and then combined with 137 cytokine-related genes from three publications in *Journal of Allergy and Clinical Immunology* [27–29]. After removing overlapping genes between the two gene sources, the cytokine-related gene set contained a total of 593 genes. The prognostic value of these genes in patient survival was calculated by the Kaplan—Meier method with the two-sided log-rank test by two packages (survival and KMsurv) of R. The permuted *P*-value for each gene was corrected by multiple comparison correction using the Benjamini—Hochberg false discovery rate (FDR). The genes with corrected permutation *P*-values < 0.01 were selected as candidate genes. In the end, six genes remained.

To evaluate the prognostic effectiveness of the six genes, a risk-score formula for predicting survival was developed based on a linear combination of the mRNA expression level (expr) weighted by the regression coefficient derived from the univariate Cox regression analysis (β) [30, 31]. The risk score for each patient was calculated as follows:

$$\text{Risk score }=\text{exp}{\text{r}}_{\text{gene}1}\times \;\beta_{\text{gene}1}+\;\text{exp}{\text{r}}_{\text{gene}2}\times \;\beta_{\text{gene}2}+\cdot \cdot \cdot +\text{ exp}{\text{r}}_{\text{genen}}\times \;\beta_{\text{genen}}$$

We next divided patients in the training dataset into high-risk and low-risk groups using the median mRNA signature risk score as the cutoff point. Patients with higher risk scores are expected to have poor survival. The same *β* was applied to the validation cohort.

Overall survival (OS) was defined as the period from the first operation to death or last follow-up. The progression-free survival (PFS) was defined as the period from the first operation to the time of tumor recurrence or evidence of progression based on magnetic resonance imaging (MRI). The differences in OS and PFS between high-risk patients and low-risk patients were estimated using the Kaplan-Meier method and 2-sided log-rank test in GraphPad Prism Version 6.01. Cox proportional hazards regression analyses were performed to assess the independent contribution of the mRNA signature and clinicopathologic variables to survival prediction.

GSEA was performed using Gene Set Enrichment Analysis v2 software downloaded from the Broad Institute (www.broadinstitute.org/gsea). The mRNA expression profile of GBM samples from the CGGA dataset was analyzed by GSEA [25, 26]. For GSEA, risk score was treated as a binary variable divided into low or high risk by a criterion of whether the score was greater than the median value. To determine detailed immunologic functional gene sets for GSEA, we used the immunologic signature gene sets from MSigDB [26]. All other parameters were set based on their default values.

Significant difference in the comparison of two experimental groups was determined by *t* test. Excel was used for correlation analysis. Principal component analysis (PCA) was conducted to assess patterns in gene expression, and PCA was conducted with the R programming language (http://cran.r-project.org). STRING, a database of protein-protein interactions, was also used. STRING takes a list of gene products and returns a diagram of known and potential interactions [32]. Chi-Square test and Fisher’s exact test were used to compare the frequencies between groups in SPSS version 13.0 software for Windows (SPSS). All differences were considered statistically significant at the level of p<0.05.

## Results

### Prediction of survival based on six cytokine-related genes signature in the CGGA database (the training cohort)

**Fig 1:**
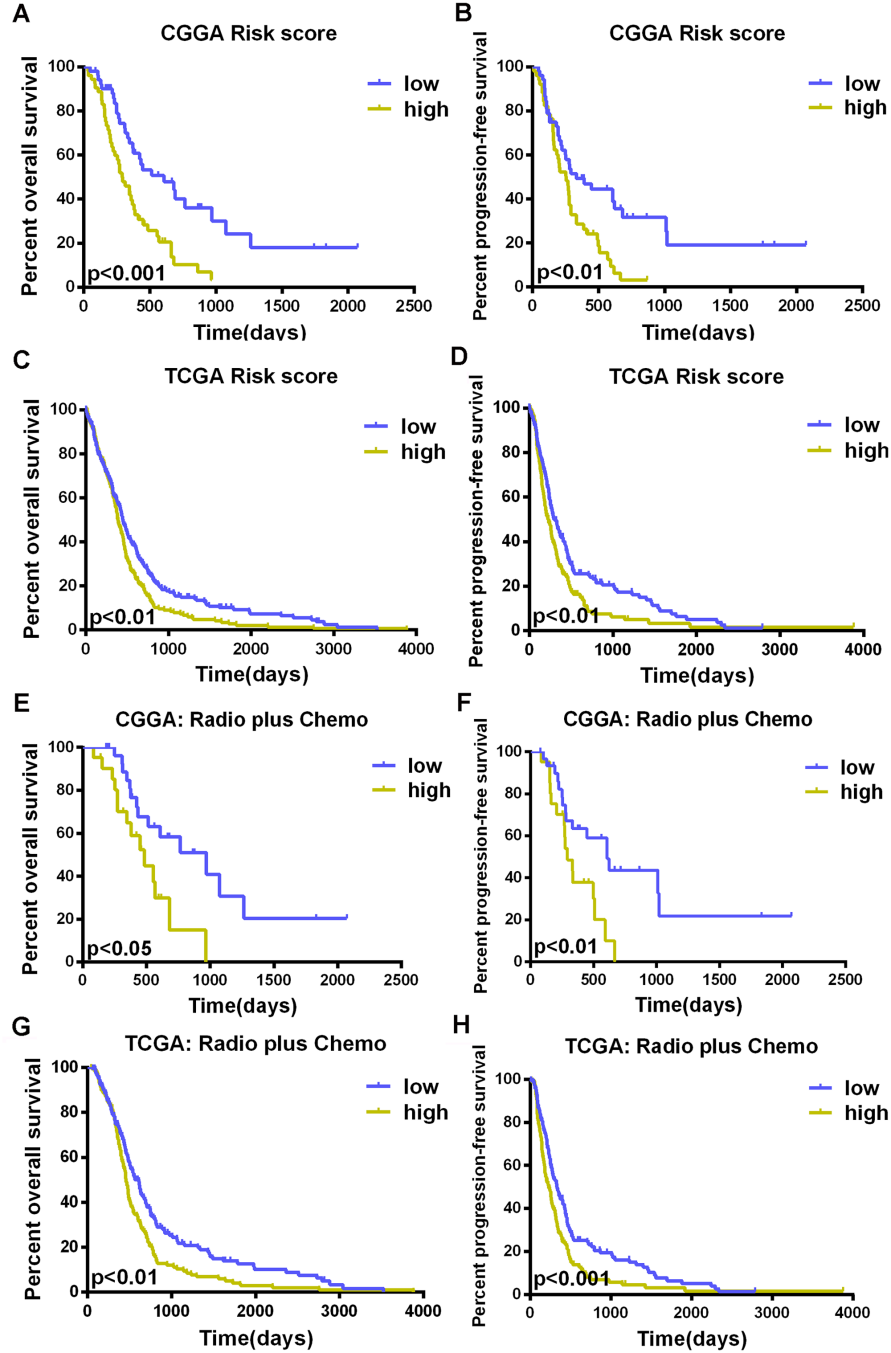
Development of the prognostic model. Kaplan—Meier curves for overall and progression-free survivals in the two groups (low risk and high risk) as defined by a prediction model based on the weighted expression of six genes (CCL2, CCR2, CXCL10, IL10RB, IL17B, and IL17R) (A, B). This Kaplan-Meier assessment of OS and PFS in patients with glioblastoma illustrates a risk score analysis using this signature in the TCGA cohort (C, D). The risk score has prognostic value of survival in the radiotherapy plus temozolomide group from both the CGGA and TCGA datasets (E, F, G, H).

In 105 CGGA primary GBM samples, we used Cox regression to analyze each of 593 cytokine-related genes in the training set and identified six genes (CCL2, CCR2, CXCL10, IL10RB, IL17B, and IL17R) that were significantly associated with OS (S1 Table, P<0.01). We then performed the risk score method to construct a model for the prediction of survival based on these six genes [30, 31]. We calculated the signature risk score for each of the 105 patients in the training set and divided them into a high-risk group (n = 52) and a low-risk group (n = 53); the median risk score was used as the cutoff point. Table 1 shows the clinical characteristics of the patients in each of the two risk groups. The rates of overall survival at one year in the low-risk and high-risk groups were 59% and 34%, respectively; the two-year survival rates were 40% and 11%, respectively. The median survival time in the low-risk group was 514 days, and that in the high-risk group was 295 days (Fig 1A, P<0.001). The high-risk score group also had shorter progression-free survival than the group with low-risk score (Fig 1B, P<0.01).

**Table 1:** Clinicopathological characteristics of patients with primary glioblastoma in the CGGA dataset (n = 105).

Variable		total	low risk score (n = 53)	high risk score (n = 52)	p value
**Age at diagnosis**	**<45**	38	22	16	>0.05
	**≥45**	67	31	36	
**Gender**	**Male**	66	32	34	>0.05
	**Female**	39	21	18	
**Preoperative KPS score**	**<80**	55	25	30	>0.05
	**≥80**	50	28	22	
	**NA**	0	0	0	
**IDH1/2 Status**	**MUT**	14	11	3	**<0.05**
	**WT**	80	35	45	
	**NA**	11	7	4	
**MGMT promoter**	**Methylation**	27	18	9	**<0.05**
**methylation**	**Unmethylation**	48	17	31	
	**NA**	30	18	12	
**Extent of surgery**	**Total**	46	21	25	>0.05
	**Subtotal**	52	27	25	
	**NA**	7	5	2	
**Radiotherapy**	**Yes**	70	39	31	>0.05
	**No**	29	13	16	
	**NA**	6	1	5	
**Chemotherapy**	**Yes**	62	35	27	>0.05
	**No**	36	16	20	
	**NA**	7	2	5	

Clinical characteristics (age, gender, pre-operational Karnofsky Performance Scale (KPS) score, and treatment) and molecular information (IDH mutation and MGMT methylation status) were obtained from the CGGA dataset and are shown in Table 1.

### Confirmation of the signature for survival prediction in the TCGA database (the validation cohort)

To validate the prognostic value of the 6-cytokine signature, we applied the risk score formulas from the CGGA dataset to patients from the TCGA dataset and then ranked the patients with known risk scores in each respective situation. These patients were also divided into low- and high-risk groups, using the median risk score as a cut-off. The prognostic value of this signature was validated in the TCGA dataset (Fig 1C and 1D).

In patients treated with radiotherapy plus temozolomide—the standard treatment for primary GBM, the risk score predicted overall survival and progression-free survival in both datasets. Patients with a high-risk score had poor survival (Fig 1E and 1F, OS, P<0.05, 484 days vs. 970 days; PFS, P<0.01, 292 days vs. 208 days; Fig 1G and 1H, OS, P<0.01, 458 days vs. 611 days; PFS, P<0.001, 233 days vs. 333 days).

**Fig 2:**
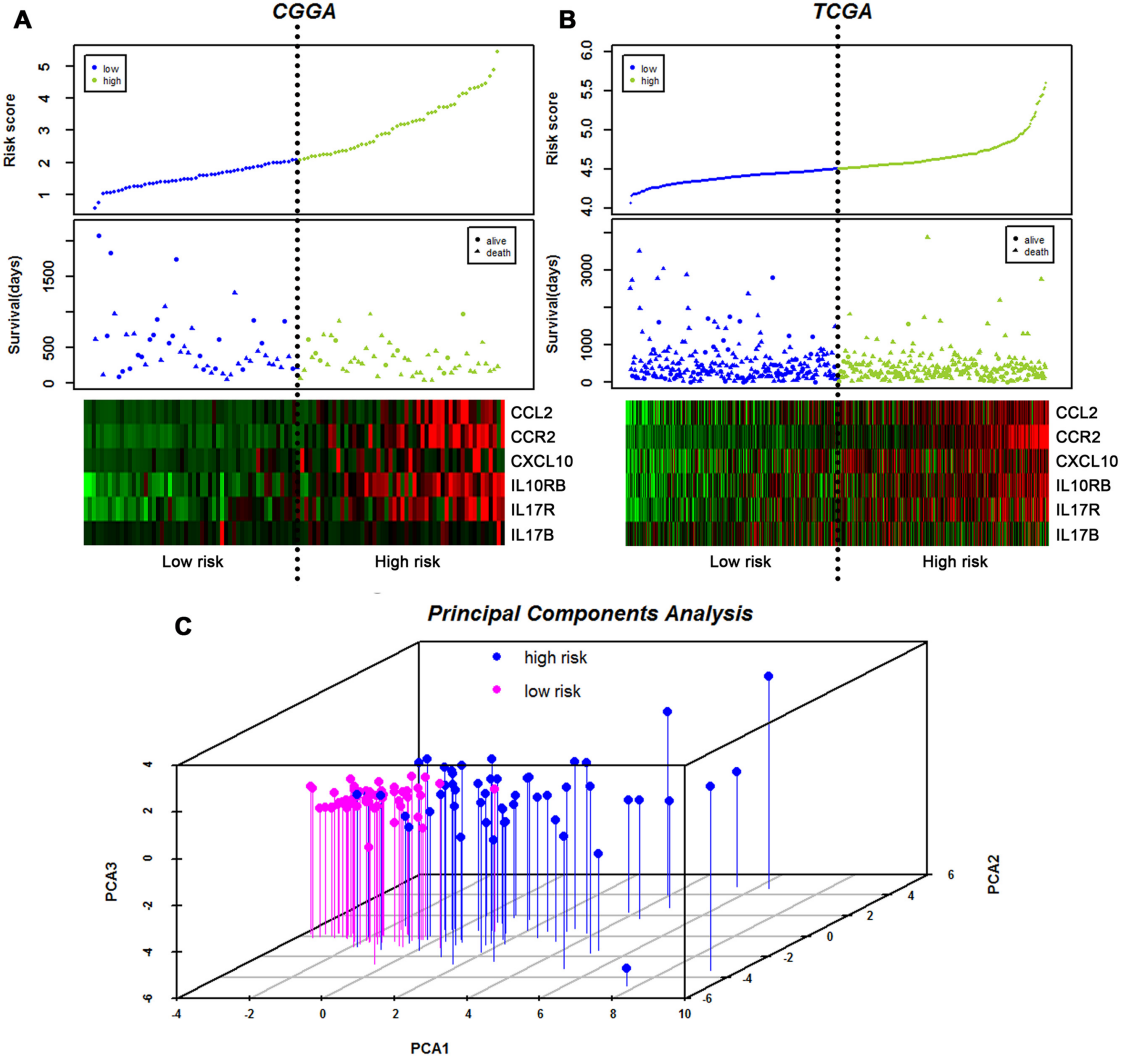
Distribution of risk scores and patient survival duration. Analysis of risk scores, OS, and mRNA expression in the CGGA cohort (A) and the TCGA cohort (B), including (Top) signature risk score distribution and (Middle) patient survival status and duration. A heat map showing expression of the six genes in both the high-risk and low-risk groups (Bottom); rows represent corresponding genes, and columns indicate corresponding patients. The black dotted lines in the middle of each graph (A, B) represent the gene signature cutoff (median risk score). Principal component analyses (C) of the CGGA dataset using the six genes. Graph of the first 3 principal components shows excellent separation between low-risk (pink) and high-risk (blue) groups. PCA1, PCA2, and PCA3 represent the top three dimensions of genes showing differential expression among these preimplantation blastomeres, which account for 41.2%, 17.6%, and 15.6% of the expressed genes, respectively.

The distribution of patient risk scores, OS, and mRNA expression in GBM for both the training set and the validation set is provided in Fig 2A and 2B. Patients with high-risk scores expressed high levels of these six genes. Principal component analysis (PCA) shows a good separation between the high-risk group and the low-risk group in the primary GBM specimens according to the six cytokine signature (Fig 2C).

### The 6-cytokine signature is an independent prognostic factor in primary GBM

We conducted univariate Cox regression analysis using clinical and genetic variables for the CGGA cohort (Table 2) and observed that the signature (risk score), radiotherapy, chemotherapy (temozolomide), pre-operative KPS score, and MGMT promoter methylation status were statistically associated with OS. We also found that risk score, chemotherapy, pre-operative KPS score, and MGMT promoter methylation status were associated with PFS. Multivariate Cox regression analysis indicated that the signature was an independent prognostic factor in the CGGA cohort (Table 2 OS: HR, 2.352; 95% CI, 1.256–4.403; P<0.01; PFS: HR, 1.78; 95% CI, 1.004–3.157; P<0.05).

**Table 2:** Factors associated with OS and PFS in the Cox regression analysis for patients from the CGGA dataset.

			Univariate Cox Regression			Mutivariate Cox Regression	
Variable		HR	95%CI	p value	HR	95%CI	p value
***Overall Survival***							
**Age at diagnosis**	**<45 vs. ≥45**	1.038	0.644–1.672	>0.05			
**Gender**	**Male vs. Female**	0.854	0.526–1.388	>0.05			
**Preoperative KPS score**	**<80 vs. ≥80**	4.869	2.886–8.216	**<0.001**	4.724	2.524–8.840	**<0.001**
**Risk score**	**Low vs. High**	2.323	1.426–3.785	**0.001**	2.352	1.256–4.403	**<0.01**
**Chemotherapy**	**Yes vs.No**	2.23	1.373–3.622	**0.001**	1.923	0.998–3.705	**0.051**
**Radiotherapy**	**Yes vs.No**	1.914	1.146–3.194	**<0.05**	0.887	0.449–1.753	>0.05
**MGMT**	**Methy vs. Unmethy**	2.088	1.175–3.710	**<0.05**	1.48	0.765–2.863	>0.05
**IDH1/2 Mutation status**	**MUT vs. WT**	1.595	0.777–3.272	>0.05			
**Extent of surgery**	**Total vs.Subtotal**	1.356	0.837–2.197	>0.05			
***Progression-free Survival***							
**Age at diagnosis**	**<45 vs. ≥45**	0.896	0.567–1.418	>0.05			
**Gender**	**Male vs. Female**	0.805	0.501–1.291	>0.05			
**Preoperative KPS score**	**<80 vs. ≥80**	4.514	2.722–7.487	**<0.001**	3.926	2.173–7.094	**<0.001**
**Risk score**	**Low vs. High**	2.019	1.259–3.239	**<0.01**	1.78	1.004–3.157	**<0.05**
**MGMT**	**Methy vs. Unmethy**	1.931	1.104–3.379	**<0.05**	1.415	0.762–2.629	>0.05
**Chemotherapy**	**Yes vs.No**	1.974	1.229–3.169	**<0.01**	1.404	0.784–2.514	>0.05
**Radiotherapy**	**Yes vs.No**	1.422	0.857–2.362	**>0.05**			
**IDH1/2 Mutation status**	**MUT vs. WT**	1.313	0.667–2.587	>0.05			
**Extent of surgery**	**Total vs.Subtotal**	1.225	0.771–1.945	>0.05			

### The high-risk score group from the 6-cytokine signature is associated with the M2 microglia/macrophage phenotype and exhibits increased expression of IL10 and TGFβ1

**Fig 3:**
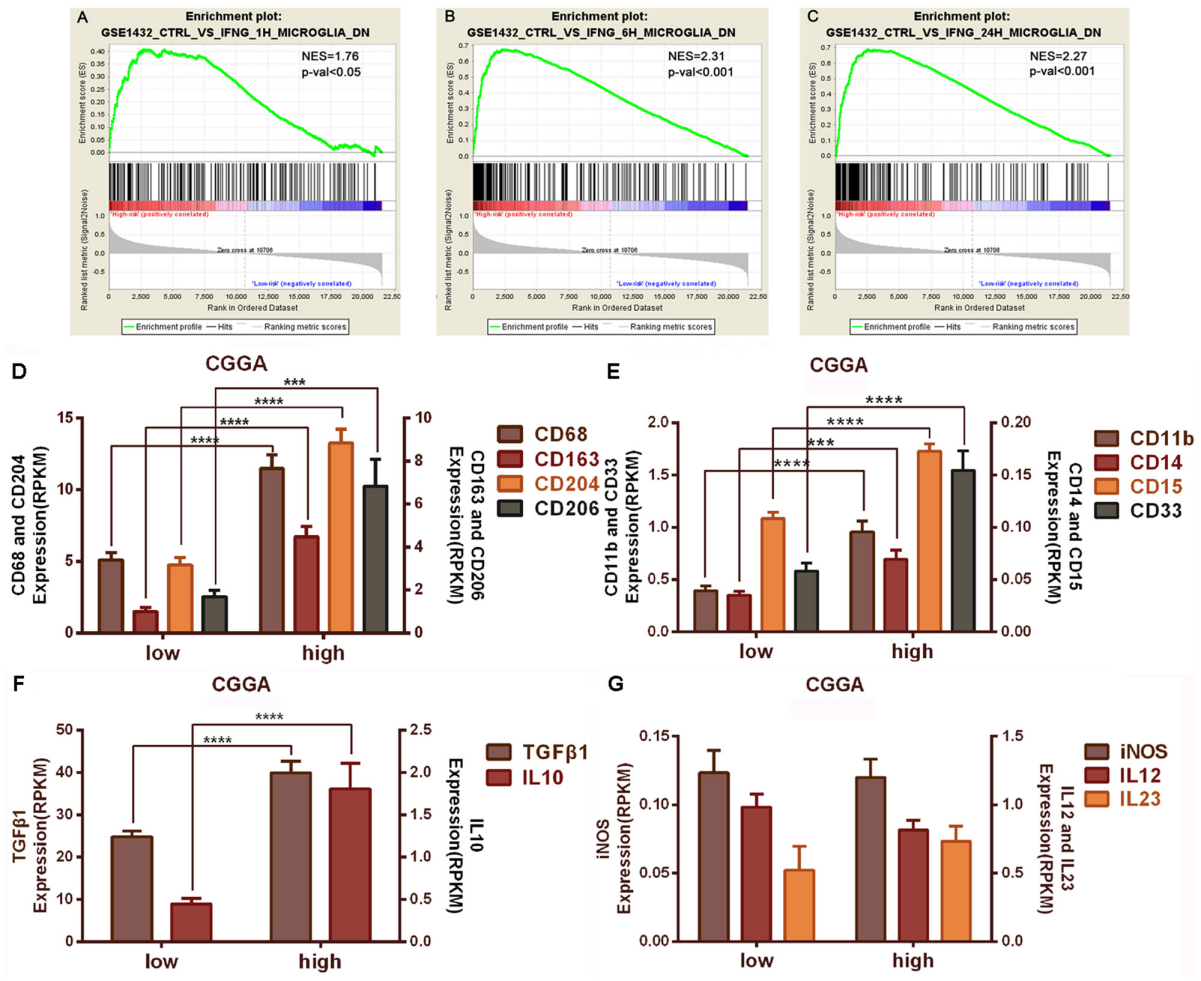
Assessment of gene expression between the high and low subgroups. The enrichment plots of the immunologic gene expression signatures of activated microglia are separated into high- and low-risk score groups. A, NES = 1.76, p-val<0.05; B, NES = 2.31, p-val<0.001; C, NES = 2.27, p-val<0.001. NES refers to Normalized Enrichment Score; p-val refers to FWER p value. M2 microglia/macrophage markers (CD68, CD163, CD204, and CD206) were significantly up-regulated in the high-risk group (D). MDSC markers (CD11b, CD14, CD15, and CD33) showed increased expression in the high-risk group (E). Genes encoding TGFβ1 and IL10 are expressed at higher levels in the high-risk group (F). M1 markers (iNOS and IL12) are not significantly different between the low-risk group and the high-risk group (G). *, P<0.05; **, P<0.01; ***, P<0.001.

To better understand the relationship between immunology and inflammation, we conducted gene expression analysis between the low-risk group and the high-risk group in the CGGA samples. Gene set enrichment analysis (GSEA) [26] revealed that the subgroup with high risk score had increased expression of activated microglia-associated genes (Fig 3A, 3B and 3C, NES = 1.76, P<0.05; B, NES = 2.31, P<0.001; C, NES = 2.27, P<0.001). Using the mRNA sequencing gene expression profile, we observed that the M2 microglia/macrophage markers (CD68, CD163, CD204, CD206) [11–13] were significantly up-regulated in the high-risk subgroup compared to the low-risk subgroup (Fig 3D, P<0.0001, P<0.0001, P<0.0001, P<0.001, respectively). Likewise, MDSC-specific transcripts (CD11b, CD14, CD15, CD33) [33] were found at elevated levels in the high-risk subgroup (Fig 3E, P<0.0001, P<0.001, P<0.0001, P<0.0001, respectively). M2 markers, CD163 and CD204, and MDSC markers, CD11b, CD14, CD15, and CD 33, showed a similar pattern of increased expression in the high-risk subgroup of the TCGA dataset (S1 Fig; CD68 and CD206 were not included in the TCGA dataset.).

Compared to the low-risk group, IL10 and TGFβ1 mRNA were significantly elevated in the high-risk score group from the CGGA dataset and the TCGA dataset (Fig 3F, S1G and S1H Fig). However, the M1 markers, iNOS and IL12, showed no significant difference between groups (Fig 3G, S1I and S1J Fig).

**Fig 4:**
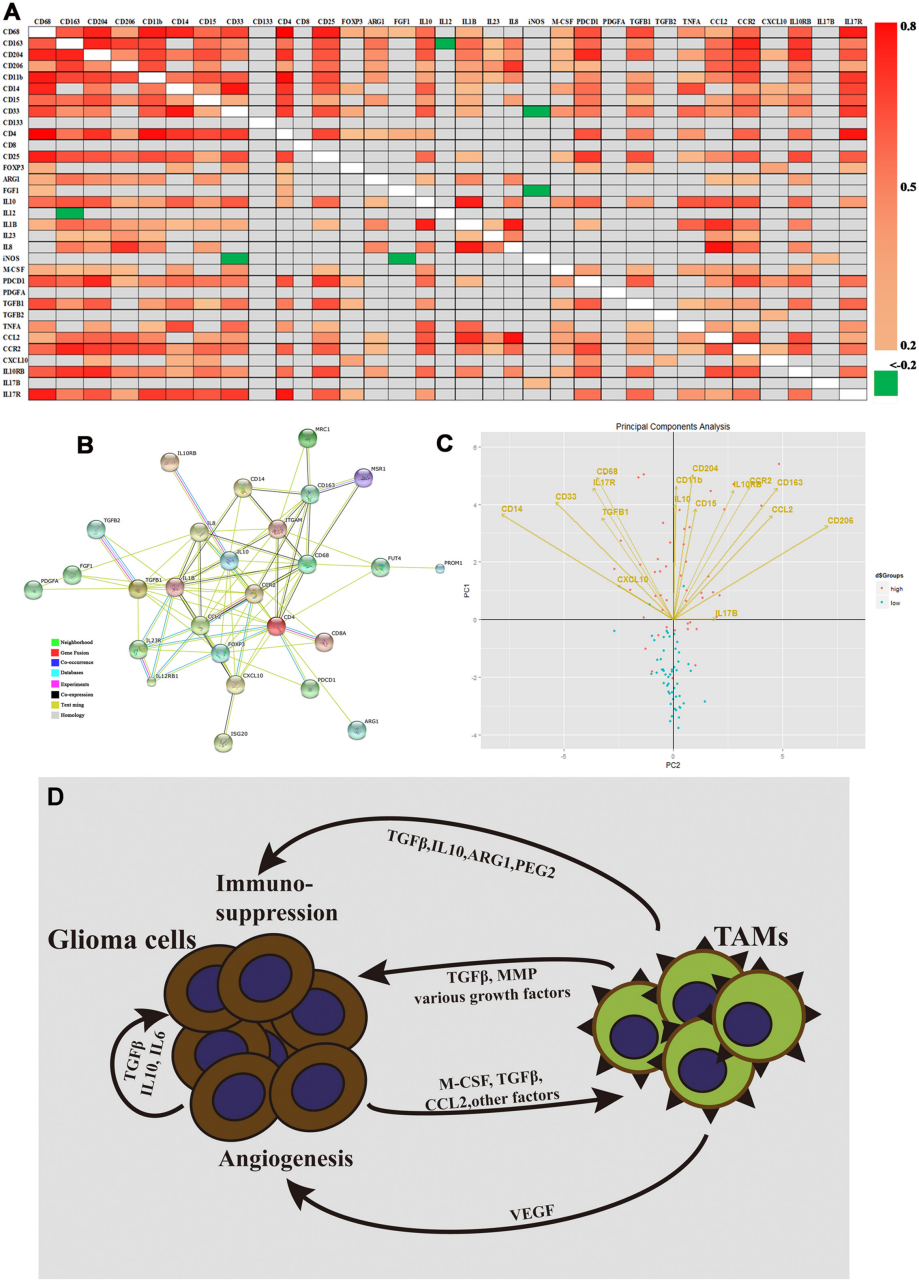
Expression patterns of cell markers and immunomodulatory genes, and Model of M2 microglia/macrophage production in GBM. (A) Heat map representation of statistically significant differentially expressed genes (P<0.05). Spearman correlation was performed between the expression levels of the indicated gene pairs in the cohort (Spearman coefficients are shown as colors corresponding to the scale bar). Gray represents gene combinations without significant correlation. (B) Protein interaction subnetwork based on protein-coding genes and their interacting partners. Nodes represent protein coding genes; links represent physical interactions. Nodes in color indicate enriched biological functions of the proteins. A red line is indicative of fusion; green line—neighborhood evidence; blue line—co-occurrence evidence; purple line—experimental evidence; yellow line—text mining evidence; light blue line—database evidence; black line—co-expression evidence. mRNA expression of genes from (C) were used for PCA analysis, which is represented by 2-dimensional visualization. The symbols represent independent patient data (blue—low risk group; pink—high risk group). PCA projections of the first 2 principal components are shown. Arrows represent individual genes with the points directed at their loading coordinates. (D) Tumor-derived molecules, such as TGFβ and M-CSF, can polarize glioma-associated microglia/microphages (MMs) toward the M2 phenotype and stimulate the production of anti-inflammatory molecules. Other glioma-derived molecules, such as CCL2 and VEGF, can recruit myeloid cells into the tumor site. TAMs refer to tumor-associated microglia/macrophages.

Using a more comprehensive panel of genes, we probed for associations between cell phenotype and immunomodulatory factors in the mRNA expression profile; results are displayed as a heat map (Fig 4A). The extent of Spearman correlation between the expression levels of the different genes was statistically significant. There was a strong association between the expression of M2 macrophage markers (CD68, CD163, CD204, and CD206) and MDSC markers (CD11b, CD14, CD15, and CD33). Additionally, expression of CCL2, CCR2, IL10RB, and IL17R positively correlated with the M2 macrophage markers and MDSC markers. Fig 4A also shows that mRNA expression of monocyte markers positively correlates with the expression of IL10 and TGFβ1. In contrast, iNOS and IL12 negatively correlate with the M2 cell marker CD163 and the MDSC marker CD33. In addition, STRING was used to visualize the gene list, containing cell markers and immunoregulatory factors; it was also used to show potential gene product interactions (Fig 4B).

PCA was conducted to determine whether or not there may be latent associations common to the genes encoding M2 (CD11b, CD68, CD163, CD204, CD206) and MDSC (CD15, CD14, CD33) phenotypic markers. The six cytokine-related genes, IL10, and TGFβ1, classical immunosuppressors, were also examined. The scores for individual samples (symbols) and the amount by which the expression of each gene "loads" on, or correlates with, the components (represented by the direction and length of the loading plot vectors) are shown in Fig 4C. The expression of genes that are near each other in the vector plots, for example, CD68, CD33, IL-17R, and TGFβ1, is expected to be associated. Moreover, there is a geometric link between the arrows and symbols indicating that higher levels of cell phenotype markers and immunoregulatory genes were significant in characterizing primary GBM samples (Fig 4C).

Consistent with previous work [34], we also observed that mesenchymal glioblastomas were enriched in the high-risk subgroups, supporting the report that immune genes exhibited differential expression in glioblastoma subtypes, and immunosuppression dominated in mesenchymal glioblastomas (S2 Table; CGGA: P<0.0001; TCGA: P<0.0001).

## Discussion

In our present work, mRNA expression profile of 105 primary GBM samples was examined by whole transcriptome sequencing, because RNA-seq can add benefits for gene expression analysis such as quantitation of transcripts, improved dynamic range, and additional capabilities for detecting expressed single nucleotide variants (SNVs), translocations, and transcript isoform switches compared with microarray and immunochemistry [15, 35, 36]. We identified a six gene cytokine-related signature (CCL2, CCR2, CXCL10, IL10RB, IL17B, and IL17R) capable of dividing glioblastoma patients into a low risk score group with favorable survival and a high risk score group with poor survival. CCL2 (MCP1) is a cognate ligand for chemokine receptor CCR2, which is expressed by monocytes in peripheral blood and plays multiple roles in cancer, including chemoattraction of circulating CCR2-positive monocytes/macrophages to the tumor vicinity [37, 38]. IL17 is a pro-inflammatory cytokine, which induces production of other pro-inflammatory cytokines, chemokines, and prostaglandins. IL17 includes six families (IL17A through F) expressed by lots of innate and adaptive immune cells, including epithelial cells, invariant natural killer T cells, natural killer cells, lymphoid-tissue inducer-like cells, neutrophils, T cells and so on[39]. In non-small-cell lung cancer patients, higher levels of IL17 within the tumor correlates with higher blood vessel density and shorter survival [40]. We have shown that expression of both IL17B and IL17 receptor are associated with survival of patients with primary GBM. Previous work has also demonstrated that endogenous CXCL10 could inhibit glioma development and promote tumor infiltration of CD8^+^ T cells in transgenic mice; additionally, myeloid-derived suppressor cells (MDSCs) produce CXCL10, indicating that they are pleiotropic [38, 41]. We found that GBM patients harboring high mRNA expression of CXCL10 had shorter overall survival in both CGGA and TCGA datasets. More work needs to be done to better understand the role of CXCL10 in glioma tumorigenesis.

We identified that the high-risk score group from the 6-cytokine signature was associated with the M2 microglia/macrophage phenotype and exhibited increased expression of IL10 and TGFβ1. These data suggested that malignant glioma cells might induce TAMs to create a favorable microenvironment for glioma progression. Microglia are abundant in the CNS and comprise approximately 5–10% (depending on the region) of all brain cells [42]. A hallmark of glioblastoma is the large number of immune cells (i.e. microglia/macrophages) that accumulate in the tumor mass [43, 44]. The number of TAMs (tumor associated microglia/macrophages) can be very high and constitute up to 30% of the tumor mass in GBM [5, 14]. Moreover, TAM number is typically higher in glioblastomas compared to grade II or III gliomas; it also closely associates with vascular density in tumors [12]. TAMs that populate gliomas can originate from either activated intrinsic parenchymal microglia or from macrophages freshly derived from bone marrow precursors in the blood [14, 45]. Peripheral blood-derived macrophages are largely restricted to the perivascular areas, meninx, and choroid plexus in the tumor-free brain; however, in GBM, these macrophages tend to assemble following the breakdown of the BBB [46]. Glioma-microglia/macrophage synergy drives a self-amplifying process in the tumor microenvironment. Glioma cells and microglial cells have a symbiotic relationship that becomes highly skewed in favor of the glioma. Glioma cells produce chemotactic factors, such as CCL2 and M-CSF, resulting in the recruitment of microglia and peripheral blood-derived macrophages. Glioma cells further promote the proliferation of microglia/macrophages. The immunosuppressive microenvironment created by molecules such as TGFβ and IL10 polarizes tumor-infiltrating microglia/macrophages toward the M2 phenotype. Lots of studies still delineated that TGFβ1 played a key role in affecting the malignant phenotype, including cell proliferation-promotion, migration and invasion-increasing, and apoptosis-inhibiton[47–52]. TGFβ1 increased glioma-initiating cells (GICs) self-renewal through the induction of LIF and the JAK-STAT pathway[53], and TGFβ inhibitors were emerging as compounds targeting GICs[54]. IL10 was associated with glioma progression. High grade gliomas expressed higher level of IL10 produced by glioma infiltrating macrophages (GIMs)[55]. IL10 was an important cytokine during glioma progression as a result that it promoted glioma invasion as well as an immunosuppressive environment[56]. In turn, M2 microglia/macrophages, influenced by the tumor itself, promote glioma growth, progression, invasion, and angiogenesis (Fig 4D) [14]. Tumor associated macrophages typically are shifted toward the M2 phenotype, tumor promoting, and immunosuppressive[57, 58]. miR-142-3p administration resulted in glioma growth inhibition by modulating M2 macrophages through the transforming growth factor beta signaling pathway[25]. Based on our result, targeting tumor-associated microglia/macrophages may be a novel, useful therapeutic approach for the high risk score group patients.

In conclusion, our signature reflects the proportion of M2 microglia/macrophages between the high-risk group and the low-risk group. The signature can be used to assess glioblastoma patient prognosis. It also provides new information regarding immunotherapy and the infiltration of immune cells into glioma tumors.

## Supporting Information

S1 Fig Confirmation of mRNA expression (cell phenotype and immunomodulatory genes) in the high and low subgroups in the TCGA dataset. CD163 and CD204 are significantly up-regulated in the high-risk group (A, B; CD68 and CD206 were not included in the TCGA dataset). Expression of CD11b, CD14, CD15, and CD33 were increased in the high-risk group (C, D, E, F). TGFβ1 and IL10 are expressed at higher levels in the high-risk group (G, H). iNOS and IL12 are not significantly different between the low-risk and high-risk groups (I, J).
(TIF)

S1 Table Six cytokines/receptors gene associated significantly with overall survival (OS). (DOC)

S2 Table Enrichment of mesenchymal glioblastomas in high risk group. (DOC)
