# Supplementary material for: Identification of a 6-Cytokine Prognostic Signature in Patients with Primary Glioblastoma Harboring M2 Microglia/Macrophage Phenotype Relevance
Source: PLoS One. 2015 May 15;10(5):e0126022. doi: 10.1371/journal.pone.0126022 (PMC4433225; doi:10.1371/journal.pone.0126022)
Supplement: S1 Table — (DOC) [file pone.0126022.s002.doc]

| **Supplementary Table S1.Six cytokines/receptors gene associated significantly with overall survival (OS)** |
| --- |

| **Symbol** | **Hazard ratio** | **95% CI** | **ParametricP-value** |
| --- | --- | --- | --- |
| **CCL2** | 1.001 | 1.0003-1.0020 | 0.009849549 |
| **CCR2** | 1.802 | 1.2115-2.6791 | 0.003641995 |
| **CXCL10** | 1.016 | 1.0060-1.0269 | 0.001976956 |
| **IL10RB** | 1.056 | 1.0146-1.0997 | 0.007734973 |
| **IL17B** | 1.182 | 1.0523-1.3288 | 0.00486366 |
| **IL17R** | 1.167 | 1.0557-1.2905 | 0.002548611 |
