# Supplementary material for: Identification of a 6-Cytokine Prognostic Signature in Patients with Primary Glioblastoma Harboring M2 Microglia/Macrophage Phenotype Relevance
Source: PLoS One. 2015 May 15;10(5):e0126022. doi: 10.1371/journal.pone.0126022 (PMC4433225; doi:10.1371/journal.pone.0126022)
Supplement: S2 Table — (DOC) [file pone.0126022.s003.doc]

| **Supplementary Table S2. Enrichemnt of mesenchymal glioblastomas in high risk group.** | | | | | | |
| --- | --- | --- | --- | --- | --- | --- |
|  |  | **Proneural** | **Neural** | **Classical** | **Mesenchymal** | **p value** |
| ***CGGA*** | **low risk** | 16 | 6 | 29 | 2 | <0.0001 |
|  | **high risk** | 0 | 2 | 7 | 43 |  |
| ***TCGA*** | **low risk** | 102 | 41 | 91 | 25 | <0.0001 |
|  | **high risk** | 35 | 42 | 52 | 130 |  |
